# Relationship Between the Frequency and Duration of Physical Therapy and Hospitalization-associated Disability Among Geriatric Patients with Heart Failure

**DOI:** 10.1298/ptr.E10283

**Published:** 2024-05-24

**Authors:** Yudai KOIWA, Shingo KOYAMA, Yuma TAKAHASHI, Kohei KAWAMURA, Yota KUNIEDA, Hiroyuki ASE, Tomomi MATSUBARA, Tadashi MIYAZAKI, Futoshi WADA, Tomokazu TAKAKURA

**Affiliations:** ^1^Department of Rehabilitation Medicine, Juntendo University Juntendo Tokyo Koto Geriatric Medical Center, Japan; ^2^Faculty of Health Science, Tsukuba University of Technology, Japan; ^3^Department of Cardiology, Juntendo University Juntendo Tokyo Koto Geriatric Medical Center, Japan; ^4^Faculty of Health Science, Juntendo University, Japan

**Keywords:** Physical therapy, Hospitalization-associated disability, Heart failure, Frequency, Duration

## Abstract

Objective: The aim of this study was to examine the relationship between the frequency and duration of physical therapy (PT) and the development of hospitalization-associated disability (HAD) in hospitalized geriatric patients with heart failure (HF). Methods: This single-center, retrospective, observational study included hospitalized patients with HF aged 65 years or older who had received PT. Data regarding demographics, comorbidities, laboratory findings, medications, rehabilitation, and activities of daily living (ADLs) status were collected from electronic medical records. Based on the average frequency and duration of PT, patients were divided into three groups: Group 1, ≥3 days/week and ≥120 minutes/week; Group 2, ≥3 days/week and <120 minutes/week; and Group 3, <3 days/week and <120 minutes/week. Logistic regression analysis was performed to identify the association between the average frequency and duration of weekly PT and the incidence of HAD. Results: In all, 105 patients (mean age, 84.8 years; proportion of women, 59%) were enrolled in the study, and 43 (41.0%) patients exhibited HAD at discharge. In the multivariate logistic regression analysis, Group 2 (odds ratio [OR], 3.66) and Group 3 (OR, 6.71) had a significantly elevated risk of developing HAD using Group 1 as the reference, even after adjusting for age, ADLs before admission, cognitive function, and severity of HF. Conclusion: This study showed that a lower frequency and shorter duration of PT are associated with developing HAD in hospitalized geriatric patients with HF. However, further prospective studies are required to confirm these findings.

## Introduction

The number of geriatric patients with heart failure (HF) is dramatically increasing in Japan. Many geriatric patients with HF have frailty, sarcopenia, cognitive impairment, and comorbidities^1)^. These patients also experience a decline in physical function and activities of daily living (ADLs) due to activity limitations and nutritional status deterioration after hospitalization^2)^.

Hospitalization-associated disability (HAD) is a serious condition that promotes poor health-related outcomes in geriatric patients with HF. This disability, which refers to new or worsened disability in ADLs during hospitalization that was not present before admission^3)^, occurs in 22.1–35.0% of hospitalized geriatric patients with medical illnesses^4^^,^^5)^. Focusing on geriatric patients with HF, the incidence of HAD is 15–41%, and HAD is a prognostic factor for health-related outcomes such as readmission and death^6^^–^^8)^. Therefore, preventing HAD in hospitalized geriatric patients with HF is important.

Rehabilitation programs during hospitalization are a strategy for preventing HAD. The Japanese Circulation Society guidelines recommend that early rehabilitation should be provided to patients with acute HF to prevent a decline in ADLs^9)^, and specifically, rehabilitation started within 3 days of hospital admission may help maintain walking ability at discharge^10^^,^^11)^. Furthermore, a recent study demonstrated that tailored progressive rehabilitation for adults aged 60 years or older with acute HF was effective in improving physical performance as measured by the Short Physical Performance Battery^12)^. These findings suggest that early rehabilitation and a tailored approach are important factors contributing to the maintenance of physical function and ADLs in geriatric patients with acute HF. However, the frequency and duration required for effective rehabilitation have not been elucidated.

Early rehabilitation programs are essential for functional recovery after hip fractures and strokes, and the amount of rehabilitation therapy required is a key factor. Physical therapy (PT) is a part of rehabilitation that includes a combination of walking, balance, and strength training to improve mobility and ADLs. Previous studies have reported that increasing the frequency and duration of PT is effective for improving function and ADLs in patients with hip fracture^13)^ and stroke^14^^,^^15)^. However, studies on the relationship between the frequency and duration of PT and ADLs in patients with HF are limited. Therefore, it is necessary to determine the different effects of frequency and duration of PT on ADLs in patients with HF. A study investigating these associations in hospitalized geriatric patients with acute HF suggested that shorter average daily rehabilitation times may increase the risk of a decline in instrumental ADLs during hospitalization^16)^. Nevertheless, no studies have investigated the relationship between the frequency and duration of PT and basic ADLs, which are widely used as a definition of HAD.

This study aimed to examine the relationship between the frequency and duration of PT and the development of HAD in hospitalized geriatric patients with HF. A systematic review showed that more frequent PT sessions with longer durations improved walking ability^17)^. Therefore, we hypothesized that both frequency and duration are related to the development of HAD. Furthermore, combining these factors would have a stronger effect on developing HAD.

## Methods

### Study design and participants

This was a retrospective, observational, single-center study. The study included patients with HF who were admitted to the Juntendo Tokyo Koto Geriatric Medical Center between April 2021 and September 2022. The inclusion criteria were patients aged 65 years or older who received PT. The exclusion criteria were as follows: death, in-hospital stroke, patients who were bedridden before hospital admission, and duration of PT treatment less than 7 days. The study was conducted in accordance with the principles of the Declaration of Helsinki. The study protocol was approved by the Ethics Committee of Juntendo Tokyo Koto Geriatric Medical Center (approval no. E22-0324-G02). Informed consent was obtained from the form on the website that included an opt-out option.

### Definition of HAD

HAD was defined, as in previous studies^6^^,^^7)^, by a decrease of at least 5 points in the Barthel index (BI) at discharge compared with that before admission. The BI is widely used to evaluate ADLs by assessing 10 items. Total scores range from 0 to 100 points, with higher scores indicating independence in performing basic ADLs. BI before admission was evaluated by asking the patient or family about the patient’s ADLs for approximately 1 month prior to hospitalization, as in previous studies^6^^,^^7)^. By contrast, the BI at discharge was evaluated by an experienced therapist based on the patient’s performance.

### Rehabilitation program

PT programs were individually designed with reference to guidelines under the supervision of a physiatrist and an experienced therapist and were carried out until discharge. The content of the PT program was composed mainly of sitting and standing practice, balance practice, muscle strengthening exercises, walking practice, ADLs practice, and aerobic exercise with a bicycle ergometer. We conducted PT programs according to the guidelines of the Japanese Circulation Society, setting the exercise intensity at a Borg Scale score of 11–13, which was adjusted for each patient’s condition and conducted step by step^9)^. PT programs were discussed for appropriateness by conference with the physiatrist and therapist. When necessary, the participants also underwent occupational or speech-language therapies. One session was performed for approximately 20–40 minutes. The frequency and duration of PT were determined by each patient’s physician and physical therapist depending on the patient’s hemodynamic status, HF symptoms, and other needs.

### Average frequency and duration of PT per week

We retrieved the average frequency and duration of PT per week from the medical records and compared them to those reported by previous studies of patients with HF, Kato et al. reported that the median length of hospital stay was 21 days, and the median number of rehabilitation days was 9 for the intermediate group^16)^. Takahashi et al. reported that the median duration of rehabilitation was 7 days, and the median total unit of rehabilitation was 15 (where 1 unit = 20 minutes)^18)^. In this study, patients were categorized into three groups based on the combination of weekly frequency and duration of PT as follows: Group 1, frequency of PT ≥3 days/week and duration of PT ≥120 minutes/week; Group 2, frequency of PT ≥3 days/week and duration of PT <120 minutes/week; and Group 3, frequency of PT <3 days/week and duration of PT <120 minutes/week. In this study, no patient had a weekly frequency of PT <3 days/week and duration of PT ≥120 minutes/week.

### Other variables

Several additional variables were investigated using medical records, including age, sex, body mass index, New York Heart Association (NYHA) class before admission, HF type, etiology, comorbidities, blood biomarkers at admission, medications at discharge, length of hospital stay, and number of days until the first PT session. The HF type was classified as HF with reduced ejection fraction (left ventricular ejection fraction [LVEF] <40%), HF with mid-range ejection fraction (LVEF 41–49%), or HF with preserved ejection fraction (LVEF ≥50%). Blood markers at admission were examined for N-terminal prohormones of brain natriuretic peptide (NT-proBNP), creatinine, blood urea nitrogen, C-reactive protein, hemoglobin, and albumin. In addition, we evaluated cognitive function using the Mini-Cog at discharge, and cognitive impairment was defined as a Mini-Cog score of <3 points based on the criteria of a previous study^19)^.

### Statistical analysis

We compared the characteristics of patients who were categorized into three groups: Group 1, Group 2, and Group 3. Differences in the variables among the three groups were analyzed using the Kruskal–Wallis test, one-way ANOVA, chi-squared test, and Fisher’s exact test. Specifically, differences in the incidence of HAD among the three groups were analyzed post hoc. Patients were then divided into two categories based on the presence or absence of HAD. Differences in variables were compared between the two groups using the paired-samples *t*-test, Kruskal–Wallis test, chi-squared test, and Fisher’s exact test. Logistic regression analysis was used to estimate the odds ratios (OR) and 95% confidence intervals (CI) of the relationships between HAD and the combination of the average frequency and duration of weekly PT in univariate and multivariate analyses. The multivariable analysis was adjusted for age, NT-proBNP, BI before admission ≥95 points, and Mini-Cog <3 points. Previous studies have represented independence with BI scores of ≥95 points^6)^ and cognitive impairment with Mini-Cog scores of <3 points^19)^. These moderator variables were selected as severity of acute illness, functional status, and cognitive status, which were reported as factors related to HAD in a previous study^20)^. Data were analyzed using the SPSS software (version 21; IBM SPSS Japan, Tokyo, Japan). A *P* value of <0.05 was considered statistically significant for all analyses.

## Results

**Fig. 1.**
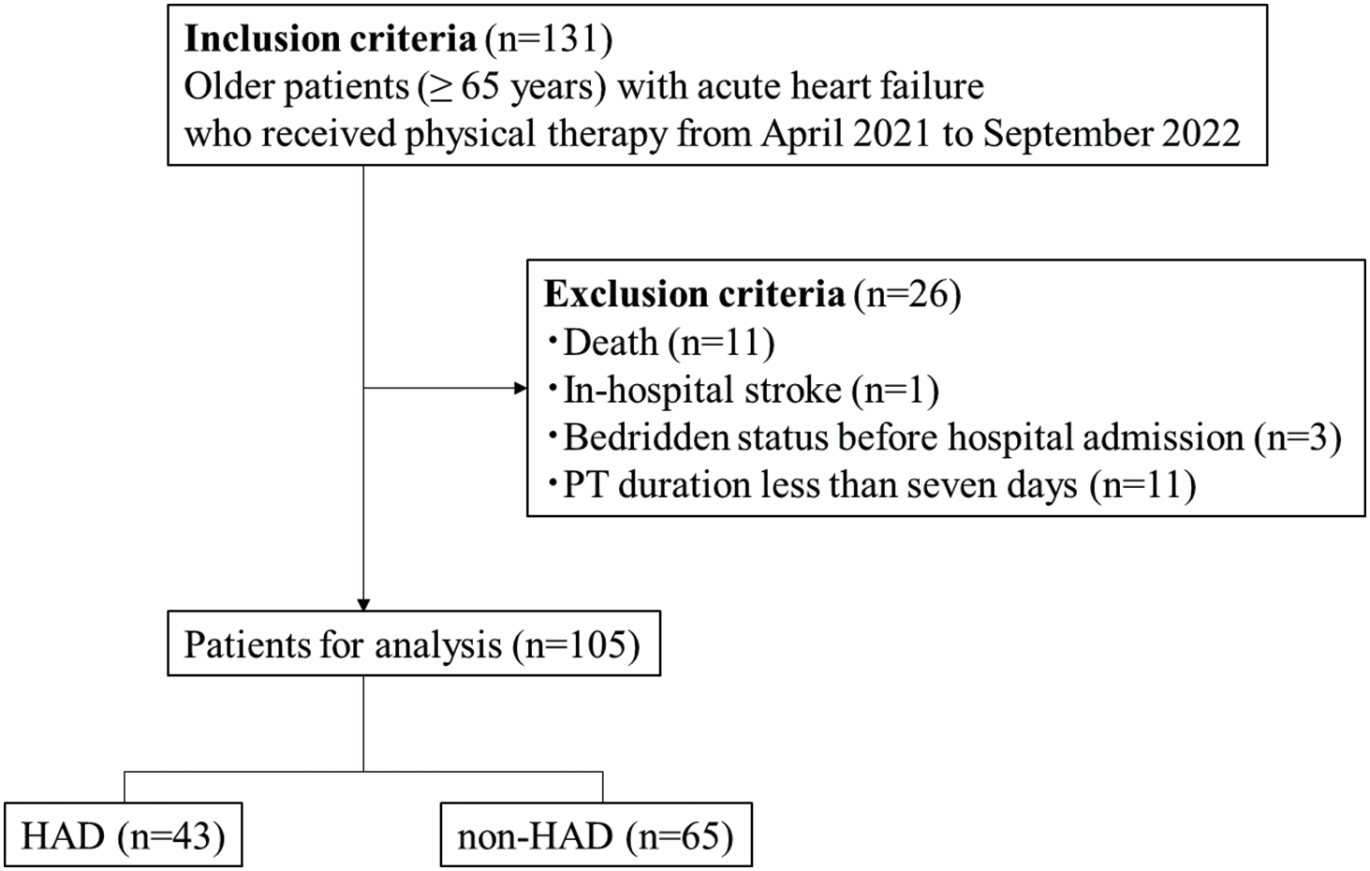
Flow chart with inclusion and exclusion criteria HAD, hospitalization-associated disability

A flowchart of the patient recruitment process is shown in Figure 1. Of the 131 patients who met the inclusion criteria, 26 were excluded because of death (n = 11), in-hospital stroke (n = 1), bedridden status before hospital admission (n = 3), or PT duration less than 7 days (n = 11). Finally, 105 patients were enrolled in the analysis, 43 (41.0%) of whom had HAD at discharge.

**Fig. 2.**
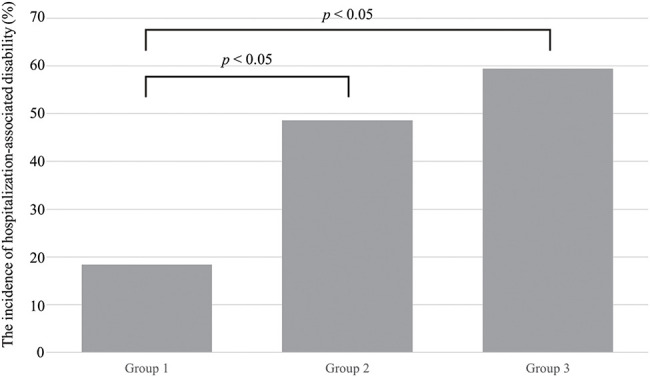
The incidence of hospitalization-associated disability among the three groups

Patient characteristics are presented in Table 1. The mean age (standard deviation) was 84.8 (5.9) years, and 62 patients (59%) were women. The median (interquartile range) BI scores were 90 (85–100) points before admission and 85 (65–100) points at discharge. Concerning all demographic and clinical characteristics mentioned in the Methods section, significant differences among the three groups were observed in NT-proBNP levels, length of hospital stay, average frequency and duration of weekly PT, BI at discharge, Mini-Cog score, and incidence of HAD (*P* <0.05). The weekly frequencies of PT in Group 1, Group 2, and Group 3 were 4.1 (3.7–4.6) days/week, 3.2 (3.1–3.5) days/week, and 2.5 (2.1–2.7) days/week, respectively. The weekly PT durations in Group 1, Group 2, and Group 3 were 143 (128–168) minutes/week, 93 (80–103) minutes/week, and 66 (52–80) minutes/week, respectively. HAD incidence in Group 1, Group 2, and Group 3 was 18.4%, 48.6%, and 59.4%, respectively. The incidence of HAD in Group 2 and Group 3 was significantly higher than that of Group 1 (*P* <0.05) (Fig. 2).

**Table 1.** Comparison of characteristics among the three groups based on the combination of the frequency and duration of weekly PT

	Overall (n = 105)	Group 1 (n = 38)	Group 2 (n = 35)	Group 3 (n = 32)	*P* value
Age (years), mean ± SD	84.8 (5.9)	83.7 (6.5)	86.1 (5.7)	84.5 (4.8)	0.208
Sex (woman), n (%)	62 (59.0)	19 (50.0)	24 (68.6)	19 (59.3)	0.272
BMI at admission (kg/m^2^), median (IQR)	21.6 (19.2–24.0)	21.8 (19.5–24.4)	21.3 (17.6–23.1)	21.8 (19.3–24.0)	0.260
NYHA class I/II/III, n (%)	2/50/53 (1.9/47.6/50.5)	1/22/15 (2.6/57.9/39.5)	1/13/21 (2.9/37.1/60.0)	0/15/17 (0/46.9/53.1)	0.390
Heart failure type, n (%)					0.802
HFpEF	64 (61.0)	24 (63.2)	22 (62.9)	18 (56.2)	
HFmrEF	20 (19.0)	6 (15.8)	8 (22.9)	6 (18.8)	
HFrEF	21 (20.0)	8 (21.1)	5 (14.3)	8 (25.0)	
Etiology, n (%)					0.986
Ischemic	25 (23.8)	9 (23.7)	9 (25.7)	7 (21.8)	
Valvular	24 (22.9)	11 (28.9)	6 (17.1)	7 (21.8)	
Myopathy	19 (18.1)	6 (15.8)	7 (20.0)	6 (18.7)	
Arrhythmia	20 (19.0)	6 (15.8)	7 (20.0)	7 (21.8)	
Others	17 (16.2)	6 (15.8)	6 (17.1)	5 (15.6)	
Comorbidities, n (%)					
Hypertension	63 (60.0)	20 (52.6)	23 (65.7)	20 (62.5)	0.492
Dyslipidemia	29 (27.6)	10 (26.3)	7 (20.0)	12 (37.5)	0.271
Diabetes	36 (34.3)	13 (34.2)	13 (37.1)	10 (31.3)	0.879
Chronic renal failure	47 (44.8)	16 (42.1)	15 (42.9)	16 (50.0)	0.773
Chronic obstructive pulmonary disease	16 (15.2)	7 (18.4)	8 (22.9)	1 (3.1)	0.064
Blood markers at admission					
NT-proBNP (pg/mL), median (IQR)	4855 (2572–12624)	3353 (2091–7813)	7843 (3442–16321)	5921 (2530–12227)	**0.036**
Cr (mg/dL), median (IQR)	1.3 (0.9–1.8)	1.4 (0.9–1.9)	1.2 (0.8–1.8)	1.3 (0.9–1.9)	0.852
BUN (g/dL), median (IQR)	28 (18–39)	29 (19–42)	23 (16–32)	28 (19–45)	0.262
CRP (mg/dL), median (IQR)	0.8 (0.2–2.1)	0.8 (0.2–1.6)	0.8 (0.3–2.3)	0.5 (0.2–1.8)	0.474
Hb (g/dL), median (IQR)	11.1 (10.0–12.3)	11.2 (10.1–12.2)	10.6 (9.8–12.3)	11.9 (10.3–12.8)	0.228
Ab (g/dL), median (IQR)	3.4 (3.1–3.7)	3.5 (3.2–3.8)	3.3 (3.0–3.6)	3.4 (3.0–3.6)	0.057
Medication at discharge, n (%)					
Beta-blocker	69 (65.7)	25 (65.8)	25 (71.4)	19 (59.4)	0.583
ARB/ACE	15/9 (14.3/8.6)	7/3 (18.4/7.9)	3/4 (8.6/11.4)	5/2 (15.6/6.2)	0.470
MRA	52 (49.5)	18 (47.4)	17 (48.6)	17 (53.1)	0.883
ARNI	25 (23.8)	11 (28.9)	7 (20.0)	7 (21.9)	0.638
SGLT2 inhibitor	13 (12.4)	5 (13.2)	4 (11.4)	4 (12.5)	0.975
Tolvaptan	49 (46.7)	20 (52.6)	16 (45.7)	13 (40.6)	0.599
Length of hospital stay (days), median (IQR)	28 (18–50)	20 (15–25)	31 (21–49)	47 (27–81)	**<0.001**
Number of days until first PT session (days), median (IQR)	6 (4–8)	6 (4–8)	6 (5–9)	7 (4–9)	0.483
Weekly frequency of PT (days), median (IQR)	3.2 (2.8–4.0)	4.1 (3.7–4.6)	3.2 (3.1–3.5)	2.5 (2.1–2.7)	**<0.001**
Weekly duration of PT (minutes), median (IQR)	100 (73–131)	143 (128–168)	93 (80–103)	66 (52–80)	**<0.001**
BI before admission (points), median (IQR)	90 (85–100)	100 (90–100)	90 (80–100)	90 (80–100)	0.055
BI at discharge (points), median (IQR)	85 (65–100)	95 (85–100)	85 (55–95)	75 (55–90)	**0.001**
ΔBI (points), median (IQR)	0 (−15–0)	0 (0–0)	0 (−15 to 0)	−5 (−25 to 0)	**0.010**
OT treatment, n (%)	6 (5.7)	1 (2.6)	3 (8.6)	2 (6.3)	0.589
SLT treatment, n (%)	16 (15.2)	4 (10.5)	8 (22.9)	4 (12.5)	0.299
Mini-Cog <3 points, n (%)	60 (57.1)	14 (36.8)	23 (65.7)	23 (71.9)	**0.006**
Hospitalization-associated disability, n (%)	43 (41.0)	7 (18.4)	17 (48.6)	19 (59.4)	**0.001**

IQR, interquartile range; SD, standard deviation; BMI, body mass index; NYHA class, New York Heart Association Class; HFpEF, heart failure with preserved ejection fraction; HFmrEF, heart failure with mid-range ejection fraction; HFrEF, heart failure with reduces ejection fraction; NT-proBNP, N-terminal prohormone of brain natriuretic peptide; Cr, creatinine; BUN, blood urea nitrogen; CRP, C-reactive protein; Hb, hemoglobin; Ab, albumin; ARB, angiotensin II receptor blocker; ACE, angiotensin-converting enzyme inhibitor; MRA, mineralocorticoid receptor antagonist; ARNI, angiotensin receptor/neprilysin inhibitor; SGLT2 inhibitor, sodium-glucose co transporter 2 inhibitor; PT, physical therapy; BI, Barthel index; OT, occupational therapy; SLT, speech-language therapy

A comparison of the characteristics of the two groups based on the presence or absence of HAD is shown in Table 2. In the HAD group, age and proportion of cognitive impairment were significantly higher (*P* = 0.024 and *P* = 0.020, respectively), the length of hospital stay was significantly longer (*P* <0.001), and BI at discharge was significantly lower (*P* <0.001) than in the non-HAD group. In addition, the HAD group had a significantly lower frequency and shorter duration of weekly PT (*P* = 0.011 and *P* <0.001, respectively) than the non-HAD group (Table 2).

**Table 2.** Comparison of characteristics between two groups based on the presence or absence of hospitalization-associated disability

	HAD group (n = 43)	Non-HAD group (n = 62)	*P* value
Age (years), mean ± SD	86.3 (5.1)	83.7 (6.1)	**0.024**
Sex (woman), n (%)	24 (55.8)	38 (61.3)	0.575
BMI at admission (kg/m^2^), median (IQR)	21.0 (18.4–23.7)	21.9 (19.5–24.4)	0.260
NYHA class I/II/III, n (%)	0/21/22 (0/48.8 /51.2)	2/29/31 (3.2/46.8/50.0)	0.458
Heart failure type, n (%)			0.062
HFpEF	26 (60.5)	38 (61.3)	
HFmrEF	12 (27.9)	8 (12.9)	
HFrEF	5 (11.6)	16 (25.8)	
Etiology, n (%)			0.401
Ischemic	12 (27.9)	13 (21.0)	
Valvular	7 (16.3)	17 (27.4)	
Myopathy	10 (23.3)	9 (14.5)	
Arrhythmia	9 (20.9)	11 (17.7)	
Others	5 (11.6)	12 (19.4)	
Blood markers at admission			
NT-proBNP (pg/mL), median (IQR)	5890 (2787–15508)	4098 (2508–1063)	0.095
Cr (mg/dL), median (IQR)	1.2 (1.0–2.2)	1.3 (0.8–1.7)	0.752
BUN (g/dL), median (IQR)	30 (16–48)	28 (25–57)	0.463
CRP (mg/dL), median (IQR)	0.5 (0.2–1.9)	0.9 (0.2–2.1)	0.237
Hb (g/dL), median (IQR)	11.1 (9.9–12.3)	11.4 (10.1–12.4)	0.752
Ab (g/dL), median (IQR)	3.4 (3.0–3.5)	3.5 (3.1–3.8)	0.156
Length of hospital stay (days), median (IQR)	44 (25–79)	22 (15–32)	**<0.001**
Number of days until first PT session (days), median (IQR)	7 (5–10)	6 (3–8)	0.151
Weekly frequency of PT (days), median (IQR)	3.0 (2.5–3.5)	3.5 (3.0–4.2)	**0.011**
Weekly duration of PT (minutes), median (IQR)	80 (57–102)	120 (84–140)	**<0.001**
BI before admission (points), median (IQR)	90 (80–100)	95 (85–100)	0.476
BI at discharge (points), median (IQR)	65 (50–80)	95 (85–100)	**<0.001**
Δ BI (points), median (IQR)	−20 (−30 to −10)	0 (0–0)	**<0.001**
OT treatment, n (%)	4 (9.3)	2 (3.2)	0.519
SLT treatment, n (%)	8 (18.6)	8 (12.9)	0.424
Mini-Cog <3 points, n (%)	30 (69.8)	30 (48.4)	**0.020**

IQR, interquartile range; SD, standard deviation; BMI, body mass index; NYHA class, New York Heart Association Class; HFpEF, heart failure with preserved ejection fraction; HFmrEF, heart failure with mid-range ejection fraction; HFrEF, heart failure with reduces ejection fraction; NT-proBNP, N-terminal prohormone of brain natriuretic peptide; Cr, creatinine; BUN, blood urea nitrogen; CRP, C-reactive protein; Hb, hemoglobin; Ab, albumin; PT, physical therapy; BI, Barthel index; OT, occupational therapy; SLT, speech-language therapy

Logistic regression analyses of HAD development are presented in Table 3. In the multivariate analysis, average frequency of weekly PT less than 3 days (OR 3.57; 95% CI 1.42–8.98) and average duration of weekly PT less than 120 minutes (OR 4.85; 95% CI 1.75–13.39) were independently associated with development of HAD, respectively. As a result of combining these factors, Group 2 (OR 3.66; 95% CI 1.19–11.24) and Group 3 (OR 6.71; 95% CI 2.10–21.40) had a significantly elevated risk of HAD development according to multivariate analysis using Group 1 as the reference.

**Table 3.** Univariate and multivariate logistic regression analyses of HAD development

	Univariate			Multivariate		
	OR	95% CI	*P* value	OR	95% CI	*P* value
Average frequency of weekly PT						
≥3 days	1.00	ref		1.00	ref	
<3 days	3.28	1.39–7.72	**0.007**	3.57	1.42–8.98	**0.007**
Average duration of weekly PT						
≥120 minutes	1.00	ref		1.00	ref	
<120 minutes	5.14	1.99–13.30	**0.001**	4.85	1.75–13.39	**0.002**
Combination of average frequency and duration of weekly PT						
Group 1 (≥3 days/week and ≥120 minutes/week)	1.00	ref		1.00	ref	
Group 2 (≥3 days/week and <120 minutes/week)	4.18	1.46–12.01	**0.008**	3.66	1.19–11.24	**0.024**
Group 3 (<3 days/week and <120 minutes/week)	6.47	2.19–19.10	**0.001**	6.71	2.10–21.40	**0.001**

OR, odds ratio; CI, confidence intervals; HAD, hospitalization-associated disabilyty; PT, physical therapy

Multivariate logistic analysis was adjusted for age, N-terminal prohormone of brain natriuretic peptide, Barthel index before admission <95 points, mini-cog <3 points

## Discussion

This study investigated the association between the average frequency and duration of PT per week during hospitalization and the incidence of HAD in geriatric patients with HF. We found that 41% of the patients had HAD and that the average frequency and duration of PT per week were independently associated with the development of HAD. Furthermore, when these factors were combined, the OR increased compared to each of the factors alone. This study suggests that a lower frequency and shorter duration of PT during hospitalization are associated with HAD development.

The incidence of HAD among the participants in this study was relatively high. Previous studies have reported that the incidence of HAD in hospitalized geriatric patients and geriatric patients with HF is 30%^21)^ and 15–41%^6^^–^^8)^, respectively. In this study, the median weekly frequency and duration of PT were 3.2 days/week and 100 minutes/week, respectively, while the median number of days until the first PT session was 6 days. Kato et al. reported that the median length of hospital stay was 21 days, and the median number of rehabilitation days was 9 for the intermediate group^16)^. Takahashi et al. reported that the median number of rehabilitation days was 7, and the median total rehabilitation unit was 15 (where 1 unit = 20 minutes)^18)^. Yagi et al. reported that the mean duration from admission to commencing rehabilitation was 9.1 days^11)^. Relative to the reports of these previous studies, the implementation of PT in this study is not significantly different. The higher incidence of HAD in the present study was attributed to the characteristics of the participants. A previous study examining the risk factors for HAD in hospitalized geriatric patients with HF reported that patients with characteristics such as age ≥80 years, female, prior stroke, low cognitive function, smoking, low ADLs before admission, high body temperature, NYHA class III–IV, low albumin, low sodium, and low estimated glomerular filtration rate were more likely to develop HAD^22)^. The participants in this prior study were characterized by older age, a higher proportion of NYHA class III–IV, low ADLs before admission, and low cognitive function. Similarly, the HAD group in this study was characterized by a higher age, proportion of cognitive impairment, and longer length of hospital stay than the non-HAD group, as in previous studies^6^^,^^16)^. Therefore, these characteristics may have enhanced the incidence of HAD among the participants in this study.

This study demonstrated that a lower frequency and shorter duration of PT are associated with developing HAD, even after adjusting for age, ADLs before admission, cognitive function, and severity of HF. The reason for this may be the lack of physical activity during hospitalization. In-hospital physical inactivity is one of the most important factors associated with developing HAD^20^^,^^23^^,^^24)^. By contrast, a randomized controlled trial examining the effectiveness of rehabilitation demonstrated that rehabilitation programs reduce the risk of HAD development in hospitalized geriatric patients with medical illnesses^25)^. Furthermore, early rehabilitation is effective in preventing HAD in geriatric patients with HF^10^^,^^11)^. The results of these studies suggest a strong relationship between physical activity during hospitalization, including rehabilitation, and the development of HAD. Therefore, in this study, we also considered whether therapeutic opportunities for PT advance physical activity during hospitalization and whether implementing PT above a certain threshold of frequency and duration would contribute to the prevention of HAD.

When categorized into three groups based on the combination of weekly frequency and duration of PT in this study, Group 2 (≥3 days/week and <120 minutes/week) and Group 3 (<3 days/week and <120 minutes/week) had a significantly higher risk for developing HAD compared to Group 1 (≥3 days/week and ≥120 minutes/week). A previous study examining the relationship between rehabilitation duration and instrumental ADLs in hospitalized geriatric patients with HF suggested that an average daily rehabilitation duration of 40 minutes or more might be beneficial for maintaining instrumental ADLs^16)^. In addition, a study involving hospitalized geriatric patients reported that high-frequency physiotherapy was an independent predictor of functional improvement^26)^. Considering these findings and the results of the present study, both the frequency and duration of rehabilitation are crucial, although the ideal length of rehabilitation to prevent disability during hospitalization remains uncertain.

The results of this study cannot explain the factors influencing the frequency and duration of PT. However, patients in Groups 2 and 3 tended to have a higher proportion of cognitive impairment and a lower BI before admission than those in Group 1. Previous studies have reported that cognitive impairment may reduce opportunities for rehabilitation^16^^,^^27)^. Furthermore, cognitive impairment and low ADLs before admission delay recovery of ADLs during hospitalization^28^^,^^29)^. Geriatric patients with HF present with various clinical symptoms such as dyspnea, cough, and precordial pain^30)^. These symptoms are not only associated with dependence on ADLs but can also frequently inhibit the implementation of rehabilitation. Therefore, for patients who cannot perform 3 days/week and 120 minutes/week of PT, identifying the underlying cause of the inability to achieve the frequency and duration of PT and targeting the cause is important, rather than merely increasing the frequency and duration of PT.

The strength of this study is that it investigated the association between the frequency and duration of PT during hospitalization and the development of HAD in hospitalized geriatric patients with HF. This study is cross-sectional, and it could not determine whether the risk of HAD was higher due to the low frequency of PT or vice versa. However, there was a significant association between the frequency and duration of PT and the development of HAD, even after statistical adjustment for several risk factors. This suggests that the implementation of PT is important. However, this study has several limitations owing to its retrospective observational design. First, the rehabilitation program was not strictly controlled for each participant. Therefore, each therapist may have had a different exercise load, and the effect of PT on HAD differed. In the future, prospective studies using unified rehabilitation programs should be conducted. Second, whether increasing the frequency or duration of PT can prevent the development of HAD in geriatric patients with HF and insufficient PT is unclear. Future studies should examine the preventive effects of increased PT on HAD. Third, we did not separately examine the BI score before admission. In this study, the frequency and duration of PT were associated with the development of HAD even after adjusting for ADLs before admission. Previous studies have reported that the factors associated with HAD varied with the degree of BI before admission^18)^. The sample was small for a separate analysis in this study, but future studies should investigate the effects of the frequency and duration of PT stratified by different BI scores before admission.

## Conclusion

The results of this study show that the average frequency and duration of weekly PT during hospitalization are associated with developing HAD in geriatric patients with HF. In particular, a lower frequency and shorter duration of PT are associated with developing HAD. These findings suggest that implementing PT above a certain level of frequency and duration would contribute to the prevention of HAD. In the future, prospective, well-designed randomized-controlled trials are needed to clarify the beneficial effects of increased PT on HAD development.

## Funding

This work was supported by the Japanese Society of Physical Therapy (JSPT024, 2022).

## Conflicts of Interest

The authors declare no conflicts of interest.
